# Quantitative lung ultrasonography: a putative new algorithm for automatic detection and quantification of B-lines

**DOI:** 10.1186/s13054-019-2569-4

**Published:** 2019-08-28

**Authors:** Claudia Brusasco, Gregorio Santori, Elisa Bruzzo, Rosella Trò, Chiara Robba, Guido Tavazzi, Fabio Guarracino, Francesco Forfori, Patrizia Boccacci, Francesco Corradi

**Affiliations:** 1 Anaesthesia and Intensive Care Unit, E.O. Ospedali Galliera, Genova, Italy; 2 Department of Surgical Sciences and Integrated Diagnostics, University of Genoa, Genoa, Italy; 3 Department of Informatics, Bioengineering, Robotics and System Engineering (DIBRIS), University of Genova, Genova, Italy; 4 Anaesthesia and Intensive Care, San Martino Policlinico Hospital, IRCCS for Oncology, Genoa, Italy; 5 Department of Clinical, Surgical, Diagnostic and Pediatric Sciences. Intensive Care Unit, Fondazione Policlinico San Matteo IRCCS, University of Pavia, Pavia, Italy; 6 Department of Anaesthesia and Critical Care Medicine, Cardiothoracic and Vascular Anaesthesia and Intensive Care, Azienda Ospedaliero Universitaria Pisana, Pisa, Italy; 7 Department of Surgical, Medical and Molecular Pathology and Critical Care Medicine, University of Pisa, Pisa, Italy; 8 Anaesthesia and Intensive Care Unit, E.O. Ospedali Galliera, Via Mura delle Cappuccine 14, Genova, Italy

**Keywords:** Pulmonary edema, Acute respiratory distress syndrome, Extravascular lung water, Computer-aided diagnosis, Lung ultrasound

## Abstract

**Background:**

This pilot study was designed to develop a fully automatic and quantitative scoring system of B-lines (QLUSS: quantitative lung ultrasound score) involving the pleural line and to compare it with previously described semi-quantitative scores in the measurement of extravascular lung water as determined by standard thermo-dilution.

**Methods:**

This was a prospective observational study of 12 patients admitted in the intensive care unit with acute respiratory distress and each provided with 12 lung ultrasound (LUS) frames. Data collected from each patient consisted in five different scores, four semi-quantitative (nLUSS, cLUSS, qLUSS, %LUSS) and quantitative scores (QLUSS). The association between LUS scores and extravascular lung water (EVLW) was determined by simple linear regression (SLR) and robust linear regression (RLR) methods. A correlation analysis between the LUS scores was performed by using the Spearman rank test. Inter-observer variability was tested by computing intraclass correlation coefficient (ICC) in two-way models for agreement, basing on scores obtained by different raters blinded to patients’ conditions and clinical history.

**Results:**

In the SLR, QLUSS showed a stronger association with EVLW (*R*^*2*^ = 0.57) than cLUSS (*R*^*2*^ = 0.45) and nLUSS (*R*^*2*^ = 0.000), while a lower association than qLUSS (*R*^*2*^ = 0.85) and %LUSS (*R*^*2*^ = 0.72) occurred. By applying RLR, QLUSS showed an association for EVLW (*R*^*2*^ = 0.86) comparable to qLUSS (*R*^*2*^ = 0.85) and stronger than %LUSS (*R*^*2*^ = 0.72)*.* QLUSS was significantly correlated with qLUSS (*r* = 0.772; *p* = 0.003) and %LUSS (*r* = 0.757; *p* = 0.005), but not with cLUSS (*r* = 0.561; *p* = 0.058) and nLUSS (*r* = 0.105; *p* = 0.744). Moreover, QLUSS showed the highest ICC (0.998; 95%CI from 0.996 to 0.999) among the LUS scores.

**Conclusions:**

This study demonstrates that computer-aided scoring of the pleural line percentage affected by B-lines has the potential to assess EVLW. QLUSS may have a significant impact, once validated with a larger dataset composed by multiple real-time frames. This approach has the potentials to be advantageous in terms of faster data analysis and applicability to large sets of data without increased costs. On the contrary, it is not useful in pleural effusion or consolidations.

**Electronic supplementary material:**

The online version of this article (10.1186/s13054-019-2569-4) contains supplementary material, which is available to authorized users.

## Introduction

Lung ultrasound (LUS) is gaining recognition as a useful tool for assessing lung pathophysiology. Lung ultrasound scores (LUSS) have been proposed for the assessment of pulmonary edema, and they have been shown to be highly correlated with extravascular lung water (EVLW) [1, 2] and pulmonary capillary wedge pressure [3] but they present some limitations. Indeed, although the recognition of B-lines and the differentiation between A- and B-pattern are simple tasks, the quantification of B-lines and the assessment of the distance between them can be difficult. This is not only when B-lines are numerous or tend to merge, but also because the mean distance between two adjacent B-lines at the lung surface should be never less than 7 mm to be significant [4]. Semi-quantitative methods have been proposed to evaluate B-lines based on visual estimation of scan percentage occupied by them [5], which is prone to eyeballing errors, or lung aeration [6], which is prone to errors due to systematic gradation of coalescent B-lines based on their presence or absence without considering their extension. Recently, a modified LUSS has been developed [7] without having been validated with a reference gold standard, i.e., invasively determined EVLW. Owing to the abovementioned difficulties in B-line quantification by current methods, it can be hypothesized that an automatic method may represent a progress in the assessment of pulmonary edema.

The objectives of this pilot study were to develop a fully automatic and quantitative scoring of the pleura involved by B-lines, for evaluating its ability to assess EVLW in comparison with the previously described semi-quantitative scores, as well as its relationship with these same scores.

## Methods

### Subjects

This was a prospective observational study of 12 consecutive patients (6 males, 6 females, age 57 ± 18 years, body mass index 22 ± 2 kg/m^2^) admitted to the intensive care unit (ICU) with acute respiratory distress syndrome (ARDS) due to septic shock (*n* = 11) or transfusion-related acute lung injury (*n* = 1). Their Simplified Acute Physiology Score was 45 ± 15, and O_2_ partial pressure-to-inspired fraction was 179 ± 81. They had a catheter positioned to assess EVLW by thermo-dilution and clinical indications for LUS. All patients were sedated with continuous infusion of propofol and mechanically ventilated with tidal volume of 6 mL/kg of predicted body weight and positive end-expiratory pressure of 5 cmH_2_O. P/F ratio was different between patients depending on severity. Although each patient was mechanically ventilated with positive end-expiratory pressure (PEEP) appropriate for the severity of lung injury, all evaluations of the ultrasound measurements were taken at 5 cmH_2_O PEEP to standardize and because high PEEP reduces the amount of vertical artifacts.

The local ethical committee approved the study (Ethics Committee for Liguria Region -n. 041/2018).

### Lung ultrasonography

Every patient underwent an ultrasound examination done by the same expert operator. Images were collected by a system (Esaote MyLab alpha or Mindray DC-N3) with a high-frequency (10 MHz), linear-array probe, while the patient was in a supine position. Transverse scanning was used to visualize the pleural line avoiding acoustic interference from the ribs. Six standard areas of each hemi-thorax were identified relative to the sternum and axillary lines: anterior, lateral, and posterior, each ones divided into upper and lower halves. On each scan, the following data were recorded by the same physician who performed lung ultrasonography: presence of A-lines, maximum number of B-lines, visual percentage of lung area occupied by confluent B-lines, visual pleural involvement > 50% or ≤50%, and tissue-like patterns (consolidations). B-lines were defined as discrete laser-like vertical hyperechoic reverberation artifacts that arise from the pleural line, extend to the bottom of the screen without fading, and move synchronously with lung sliding. B-lines were defined coalescent when they are numerous, tend to merge, and impossible to clearly enumerate [8]. The most pathological pattern acquired in each intercostal space during an entire respiratory cycle was retained for the further analysis and considered representative of the area score. Five scores were defined as follows: (1) maximum number of B-lines detected (nLUSS), (2) visual percentage of lung area occupied by B-lines (%LUSS) [5], (3) B-line coalescence (cLUSS) [6], (4) modified B-line coalescence score (qLUSS) [7], (5) computer-aided score (QLUSS).

For nLUSS, the total number of B-lines seen in an intercostal space over an entire respiratory cycle was counted, with fused B-lines being counted as a single B-line.

For %LUSS, fused B-lines were counted as the percentage of the rib space filled with confluent B-lines divided by 10 and added to any other B-line noted in the space at the same time, with white lung pattern counted as 10 B-lines.

For cLUSS, the regional aeration of each examined region was graded between 0 and 3, depending on the amount of aeration loss, with 0 corresponding to A-lines or < 2 B-lines, score 1 to ≥ 3 for spaced B-lines, score 2 to coalescent B-lines, and score 3 corresponding to the presence of tissue-like pattern.

For qLUSS, coalescent B-lines were scored 1 if occupying ≤ 50% and 2 when > 50% of the intercostal space.

**Fig. 1:**
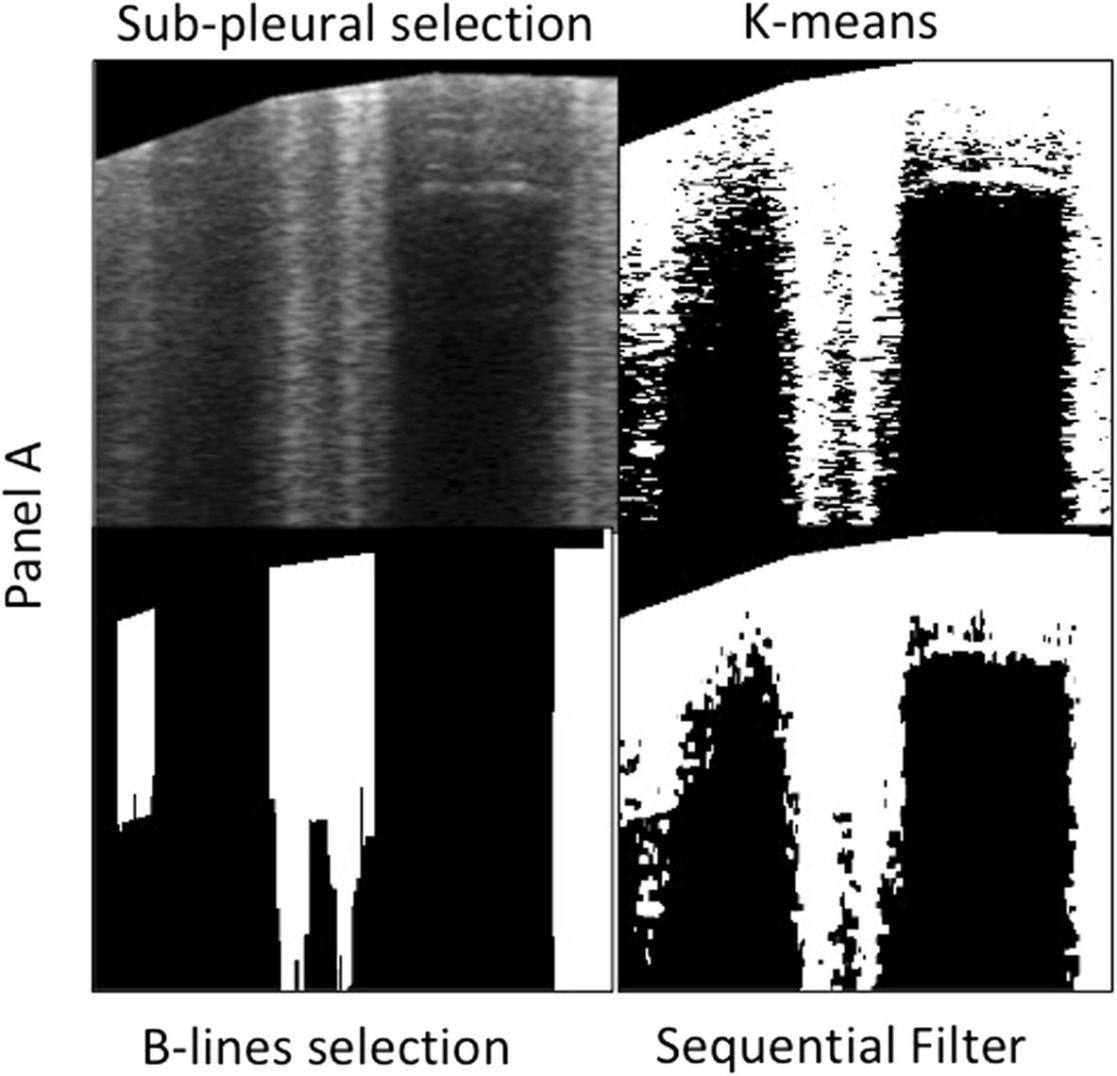
Segmentation algorithm basic steps. *Upper left*: sub-pleural selection from the original ultrasound scan. *Upper right*: K-means classification to divide pixels into two subsets. *Bottom right*: alternated sequential filter consisting in iterative morphological openings and closings, with an axial-line structuring element which closes small (10-pixel) gaps axially and isolates the objects laterally, thus enhancing the B-line contours. *Bottom left*: B-line detection scanning the images along their columns selecting those with ≥ 50% of white pixels over 70% of their total vertical length

For QLUSS, an automated scoring algorithm was used. Gray-scale images, acquired with linear probes, were imported in MATLAB and analyzed by a dedicated segmentation algorithm consisting of these sequential steps applied to every frame: (1) contrast adjustment to maximize resolution; (2) K-means classification to divide pixels into two subsets; (3) an alternated sequential filter (ASF) consisting in iterative morphological openings and closings, with an axial-line structuring element which closes small (10-pixel) gaps axially and isolates the objects laterally, thus improving B-line outlines; (4) scanning of the resulting images along their columns selecting those with ≥ 50% of white pixels over 70% of their total vertical length (Fig. 1); (5) QLUSS computation for each patient, given by the intra-patient mean percentage of B-line area of all frames.

To assess inter-rater variability, single frames were randomly distributed to two physicians expert in lung ultrasonography for off-line B-line scoring. Pairwise, the single frames were distributed to two bioengineers to do QLUSS analysis. All the raters were blinded to the patients’ conditions and clinical history.

### Thermo-dilution

A method previously described was used to determine EVLW [3].

### Statistical analysis

The study was powered to detect the correlations between the LUS scores. By assuming *r* = 0.8 (alpha = 0.05, *n* = 12, two-sided alternative hypothesis), we obtained a statistical power = 0.924. With *r* = 0.75 (alpha = 0.05, *n* = 12, two-sided alternative hypothesis), statistical power = 0.85. Results are reported as numbers, percentages (%), mean ± SD, and/or 95% confidence interval (CI). The Shapiro-Wilk test was used to evaluate normality distribution of continuous variables. Correlations between LUS scores were evaluated by the Spearman rank test. Comparisons between correlation coefficients were performed by using Hittner, May, and Silver’s modification of Dunn and Clark’s *z* [9]. The association between LUS scores and EVLW was evaluated by simple linear regression (SLR) and several robust linear regression (RLR) methods [Huber loss, least mean squares (LMS), least trimmed squares (LTS), least absolute deviation (LAD), S estimator, MM estimator]. Inter-observer variability was tested by intraclass correlation coefficient (ICC) in two-way models for agreement (strength of absolute agreement between raters was considered poor, fair, moderate, strong, or almost perfect according to ICC values < 0.30, 0.3–0.49, 0.50–0.69, 0.70–0.89, and ≥ 0.90, respectively). Statistical significance was assumed for *p* < 0.05 with a two-tailed null hypothesis. Statistical analyses were performed using software package SPSS (version 20.0; SPSS Inc., Chicago, IL, USA) and R statistical environment (version 3.5.3; R Foundation for Statistical Computing, Vienna, Austria) with the cocor package [10].

## Results

Twelve intercostal spaces of each patient were examined, providing 144 single frames analyzed. A-lines or < 2 B-lines were visualized in 24 scans, three or more well-spaced B-lines in 25 scans, coalescent B-lines in 84 scans, coalescent B-lines occupying > 50% of intercostal space in 10 scans, and tissue-like pattern in 11 scans.

**Fig. 2:**
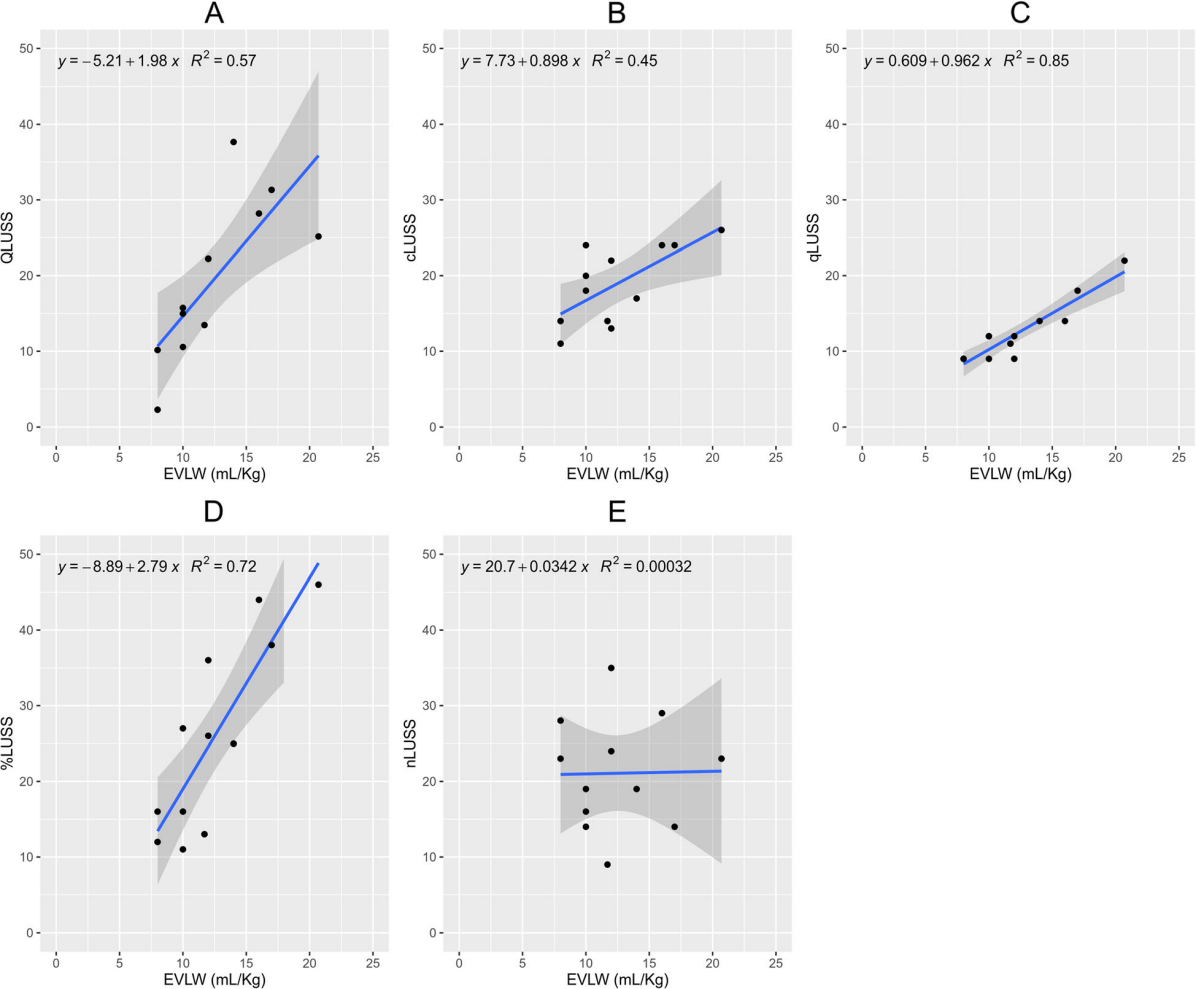
Simple linear regression models by entering the semi-quantitative scores [B-line coalescence score (cLUSS), modified B-line coalescence score (qLUSS), percentage of lung area occupied by B-lines (%LUSS), maximum number of B-lines (nLUSS)] and quantitative computer-aided score (QLUSS) for evaluating their association with extravascular lung water (EVLW). **a** QLUSS~EVLW (*p* = 0.005). **b** cLUSS~EVLW (*p* = 0.016). **c** qLUSS~EVLW (*p* < 0.001). **d** %LUSS~EVLW (*p* < 0.001). **e** nLUSS~EVLW (*p* = 0.956)

**Fig. 3:**
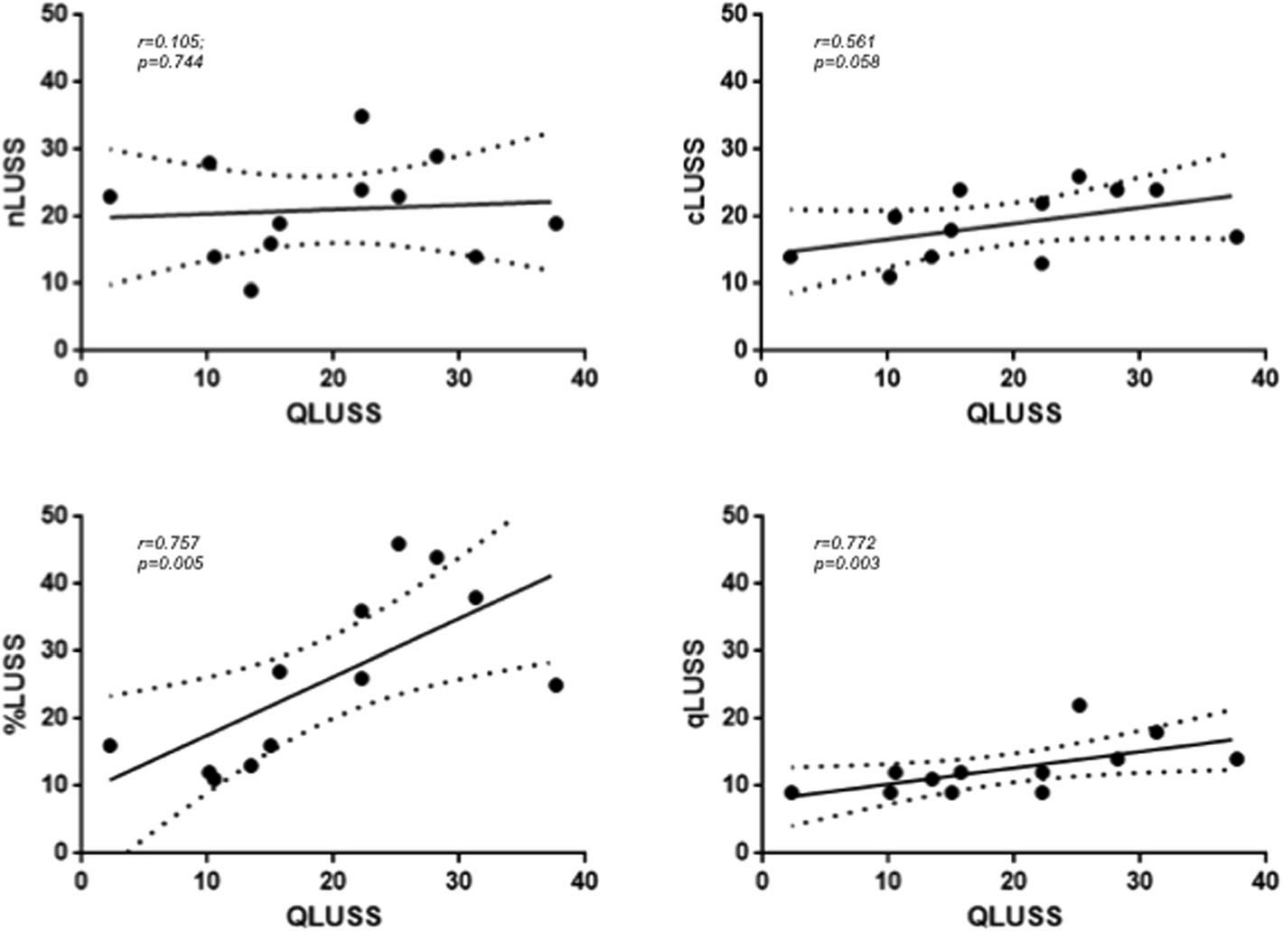
Correlations of computer-aided score (QLUSS) with semi-quantitative scores [maximum number of B-lines (nLUSS), visual percentage of lung area occupied by B-lines (%LUSS), B-line coalescence score (cLUSS), modified B-line coalescence score (qLUSS)] in 12 mechanically ventilated patients

The LUS scores rated in each patient are presented in Table 1. In the SLR, QLUSS showed a stronger association with EVLW (*R*^*2*^ = 0.57) than cLUSS (*R*^*2*^ = 0.45) and nLUSS (*R*^*2*^ = 0.000), while a lower association with EVLW than qLUSS (*R*^*2*^ = 0.85) and %LUSS (*R*^*2*^ = 0.72) occurred (Fig. 2). In the RLR, QLUSS showed an association with EVLW (*R*^*2*^ = 0.86) comparable to qLUSS (*R*^*2*^ = 0.85) and stronger than %LUSS (*R*^*2*^ = 0.72) by applying the LTS and LMS methods (for more details, see Additional file 1). QLUSS was significantly correlated with qLUSS (*r* = 0.772; *p* = 0.003) and %LUSS (*r* = 0.757; *p* = 0.005), but not with cLUSS (*r* = 0.561; *p* = 0.058) and nLUSS (*r* = 0.105; *p* = 0.744) (Fig. 3). By comparing the *r* coefficients, a significant difference occurred for nLUSS/QLUSS vs. %LUSS/QLUSS (*p* = 0.037), while almost significance was found for qLUSS/QLUSS vs. nLUSS/QLUSS (*p* = 0.071).

**Table 1:** The LUS scores and the EVLW values for each patient enrolled in this study

Patients	QLUSS	qLUSS	%LUSS	cLUSS	nLUSS	EVLW (mL/kg)
1	2.26	9	16	14	23	8.00
2	15.72	12	27	24	19	10.00
3	31.31	18	38	24	14	17.00
4	15.00	9	16	18	16	10.00
5	22.24	12	26	22	35	12.00
6	28.19	14	44	24	29	16.00
7	25.17	22	46	26	23	20.70
8	37.66	14	25	17	19	14.00
9	13.46	11	13	14	9	11.70
10	22.21	9	36	13	24	12.00
11	10.15	9	12	11	28	8.00
12	10.55	12	11	20	14	10.00

Absolute agreement between raters was strong for cLUSS [ICC 0.703 (95%CI from 0.303 to 0.858)], %LUSS [ICC 0.745 (95%CI from 0.631 to 0.822)], qLUSS [ICC 0.825 (95%CI from 0.705 to 0.896)], and nLUSS [ICC 0.895 (95%CI from 0.835 to 0.931)], with the best output for QLUSS [ICC 0.998 (95%CI from 0.996 to 0.999)].

## Discussion

The main findings of this study are that the new B-line scoring system QLUSS (1) is associated with EVLW better than cLUSS or nLUSS, returning comparable or better association than qLUSS or %LUSS; (2) is correlated with qLUSS and %LUSS but not cLUSS or nLUSS; and (3) showed the best inter-observer agreement among the different scores.

**Fig. 4:**
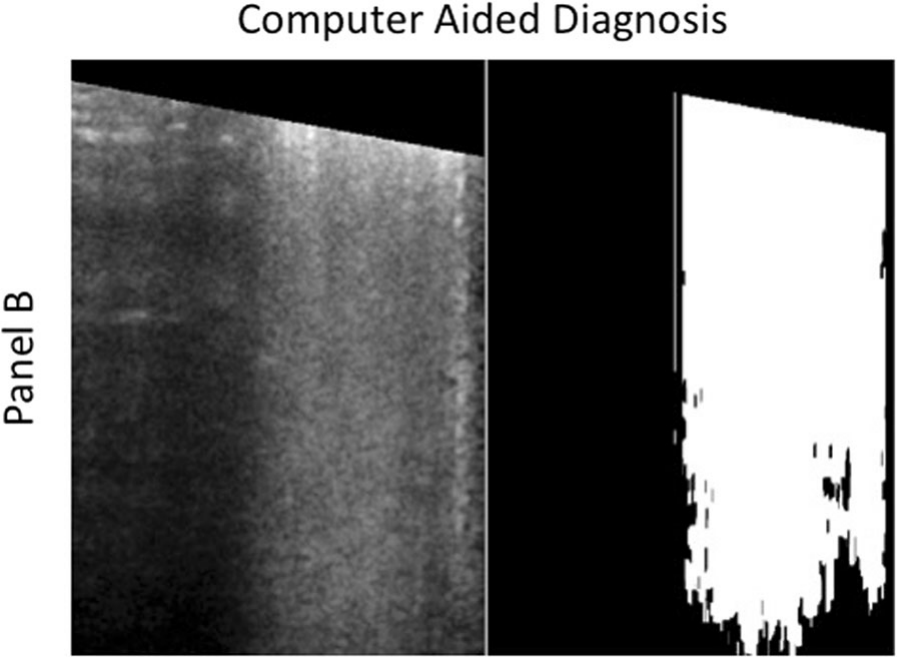
*Left*: involvement of about half of the pleural line, difficult to be objectively quantified by the eye. *Right*: computer-aided QLUSS showing an involvement of the pleural line by 40%

In a number of previous studies, LUS was used to detect and quantify pulmonary edema [3, 11]. However, LUS has limitations due to the difficulty of counting every single artifact on moving images over the whole chest. Indeed, sometimes B-lines can be easily enumerated if they are few, but most of the times they are numerous and tend to merge, making it impossible to enumerate them correctly. Therefore, for a more comprehensive estimation of EVLW, it is now recommended to assess the percentage of pleural line occupied by B-lines, rather than counting the maximum number of B-lines over each ultrasound scan [5]. However, at present, no tool is available to quantify the percentage of pleural line occupied by B-lines, which may not be a simple cognitive process. Regarding the scores proposed, cLUSS, which is mainly based on the recognition and differentiation between well-spaced B-lines or coalescent B-lines, has at least three weaknesses. First, the distance between two B-lines may not be reliably assessed visually and the concept of “well-spaced” is not easily identified. Second, also “coalescence” may be difficult to assess because considering an artifact as two close B-lines or a wide B-line is mainly a matter of individual judgment. Third, rating all coalescences with the same score, regardless of the percentage of pleura involved, leads to overestimation of EVLW when this is focal, as in ARDS. qLUSS was introduced to obviate the latter pitfall of cLUSS, thus rating with a higher score only coalescences involving > 50% of the visualized pleura. Nevertheless, also an involvement of the pleural line by more or less than 50% can be difficult to be visually recognized (Fig. 4), especially in the presence of multiple coalescent B-lines with spared areas in between. This is likely the reason why in the present study qLUSS performed better than cLUSS, %LUSS, and nLUSS, but not better than QLUSS when evaluated in RLR for association with the gold standard EVLW.

This study has some limitations. First, the study was conducted in a limited number of patients monitored for pulmonary congestion by EVLW. Larger studies are needed for clinical validation. Second, the scoring analyses was performed on single frames, and an implementation of our software to multi-frame analysis is the planned as a next step in order to analyze the entire respiratory cycle and minimize operator biases due to frame selection. Third, the computer-aided analysis (QLUSS) was performed in post-processing on single frames previously stored and future implementation on ultrasound machines might allow a real-time analysis. Fourth, QLUSS is not useful in pleural effusion or consolidations.

## Conclusions

This pilot study shows that computer-aided measurement of the percentage of pleural line affected with B-line artifacts can provide a reliable operator-independent assessment of extravascular lung water in ARDS, which seems to be comparable or superior to previously described scores. This approach has the potentials to be advantageous in terms of faster data analysis and applicability to large sets of data without increased costs.

## Additional file

Additional file 1: Electronic Statistical Supplementary Material. (DOCX 89 kb)

## Data Availability

The dataset analyzed during the current study is available from the corresponding author on reasonable request.

## Abbreviations

ARDS: Acute respiratory distress syndrome
ASF: Alternated sequential filter
CI: Confidence interval
EVLW: Extravascular lung water
ICC: Intraclass correlation coefficient
ICU: Intensive care unit
LUS: Lung ultrasound
LUSS: Lung ultrasound scores
