# Supplementary material for: Quantitative lung ultrasonography: a putative new algorithm for automatic detection and quantification of B-lines
Source: Crit Care. 2019 Aug 28;23:288. doi: 10.1186/s13054-019-2569-4 (PMC6712728; doi:10.1186/s13054-019-2569-4)
Supplement: Supplementary file 1 — Electronic Statistical Supplementary Material. (DOCX 89 kb) [file 13054_2019_2569_MOESM1_ESM.docx]

**Electronic Supplementary Material**

**Quantitative Lung Ultrasonography: a new algorithm**

**for automatic detection and quantification of B-lines**

Claudia Brusasco, Elisa Bruzzo, Rosella Trò, Gregorio Santori, Chiara Robba, Guido Tavazzi, Fabio Guarracino, Francesco Forfori, Patrizia Boccacci, Francesco Corradi

**1. Evaluation of the continuous variables for normal distribution**

The continuous variables were preliminarily evaluated for their distribution by using the Shapiro-Wilk test for normality. The results are reported in the Table ESM 1.

**Table ESM 1.** Results of the Shapiro-Wilk normality test.

| **Variable** | **W** | **P value** |
| --- | --- | --- |
| EVLW | 0.914 | 0.241 |
| QLUSS | 0.983 | 0.991 |
| cLUSS | 0.923 | 0.308 |
| qLUSS | 0.837 | *0.025* |
| nLUSS | 0.980 | 0.984 |
| %LUSS | 0.904 | 0.180 |

If the *P* value of the Shapiro-Wilk test is greater than 0.05, the data is normally distributed. If it is below 0.05, the data significantly deviate from a normal distribution. In our series, qLUSS reached statistical significance with the Shapiro-Wilk test, suggesting that for this variable the data are not normally distributed (density plots are reported in **Figure ESM 1**). These findings raise concerns about the possibility to use parametric Pearson’s correlation, by suggesting to use non parametric Spearman rank correlation. The outcomes of the Spearman correlation between semi-quantitative scores (nLUSS, %LUSS, cLUSS, qLUSS) and the proposed quantitative scoring system of B-lines (QLUSS) are reported in the article (see “Results” section and Figure 3).


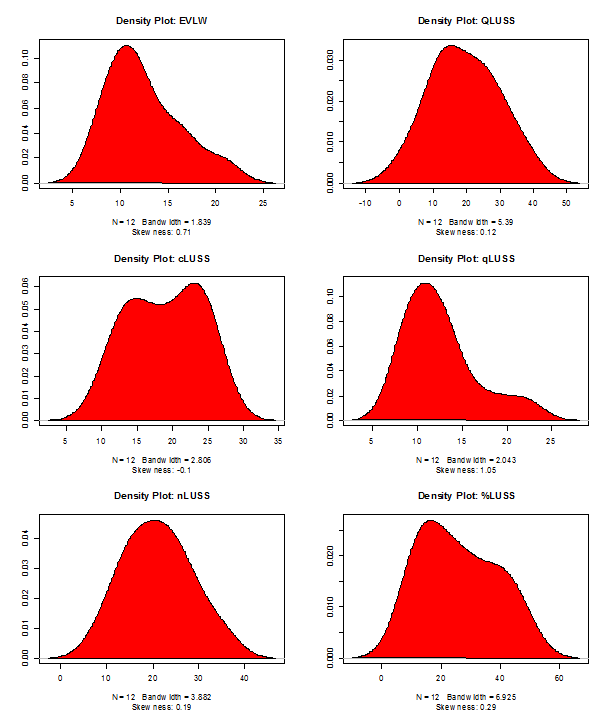


**Figure ESM 1**. Density plots for EVLW, the quantitative score (QLUSS), and the

semi-quantitative scores (cLUSS, qLUSS, nLUSS, %LUSS).

**2. Evaluation of the association between the LUS scores and EVLW**

The association between the LUS scores and EVLW was evaluated by simple linear regression (SLR) and robust linear regression (RLR). The full outcomes of the SLR are presented in the article (see “Results” section and Figure 2). By applying SLR, we found that qLUSS and %LUSS had a stronger association with EVLW than QLUSS. On the other hand, QLUSS had a stronger association with EVLW than cLUSS and nLUSS. However, in several linear regression model we noted the presence of one or more outliers. Finally, qLUSS was the only score that did not pass the Shapiro-Wilk test for normality, while QLUSS returned the best W and P value. Considering the low numerosity of our series and the presence of outliers, the robust linear regression methods should be used.

By using the SLR model A (QLUSS ~ EVLW) as example, we found an outlier in the diagnostic Cook’s bar plots (**Figure ESM 2**), also confirmed for the SLR models B (cLUSS ~ EVLW), D (%LUSS ~ EVLW) and E (nLUSS ~ EVLW).


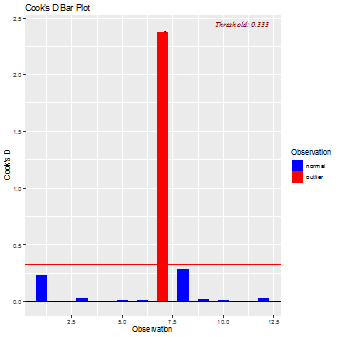


**Figure ESM 2**. Cook’s bar plot for the linear regression model A (QLUSS ~ EVLW).

For the SLR model A (QLUSS ~ EVLW), the plots to measures the difference in each parameter estimate with and without the influential point are reported in **Figure ESM 3**.


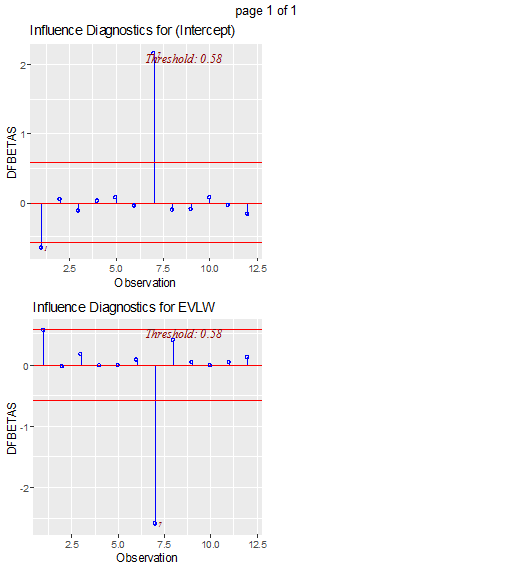


**Figure ESM 3**. Difference in each parameter estimate with and without the influential point for the model A (QLUSS ~ EVLW).

A more detailed plot for detecting influential observations of the model A (QLUSS ~ EVLW) is reported in the **Figure ESM 4**.


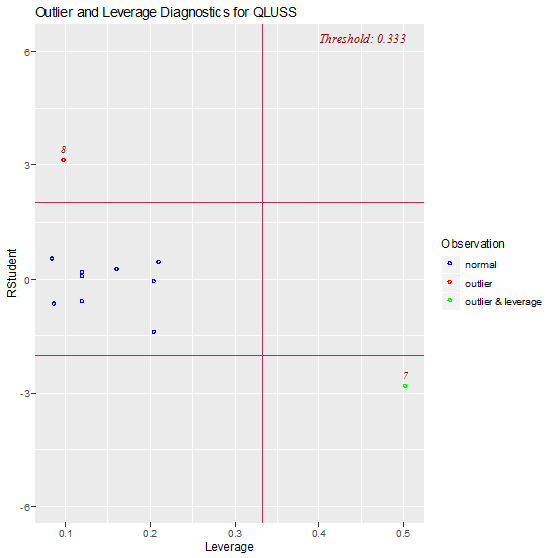


**Figure ESM 4**. Outlier and leverage diagnostics for QLUSS in the linear regression model A (QLUSS ~ EVLW).

The Figure ESM 4 suggests to evaluate also the studentized deleted residuals (the deleted residuals divided by their estimated standard deviation) (**Figure ESM 5**).


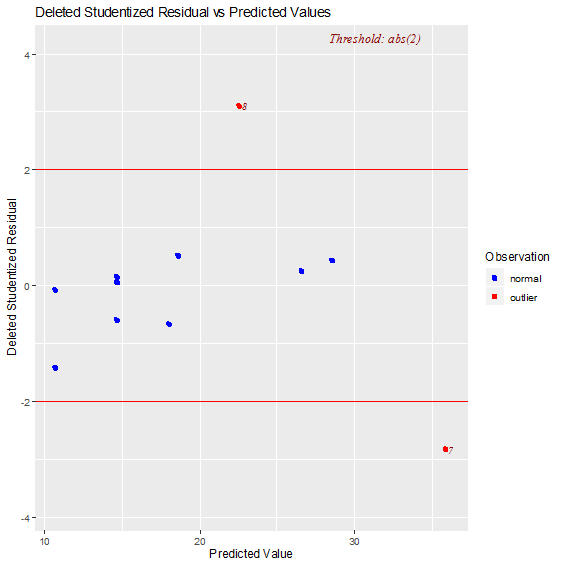


**Figure ESM 5**. Deleted studentized residual vs. predicted values of the linear regression

model A (QLUSS ~ EVLW).

In view of the previous diagnostic results for the majority of SLR models, we tested several robust linear regression (RLR) methods [Huber loss, least mean squares (LMS), least trimmed squares (LTS), least absolute deviation (LAD), S-estimator, MM-estimator] in comparison with SLR. For model A (QLUSS ~ EVLW), all methods are robust except for the SLR, the Huber loss and the MM-estimator (**Figure ESM 6**). The LTS and LMS methods increase R^2^ of the model from 0.5668522 (SLR; 0.57) to 0.8590162 (0.86), with an improvement of 50.87%. Interestingly, in the model C (qLUSS ~ EVLW) the LTS or LMS methods did not increase R^2^ (0.8489125 in both SLR and RLR). This finding was observed also in the model D (%LUSS ~ EVLW; R^2^ = 0.7176788 in both SLR and RLR). Thus, considering our low-numerosity series, QLUSS produced the best result by applying RLR methods (LTS and LMS), with an R^2^ comparable to qLUSS and slightly higher than %LUSS. Conversely, the SLR returned higher R^2^ for qLUSS and %LUSS than QLUSS, while QLUSS showed a stronger association with EVLW than cLUSS and nLUSS. The RLR should be preferred offering more protection from the outliers, which play a key role in the presence of small samples.

**Figure ESM 6**. Evaluation of simple linear regression (SLR) *vs*. several robust linear regression methods (LAD: least absolute deviation; LTS: least trimmed squares; LMS: least mean squares)

for model A (QLUSS ~ EVLW).
